# The Library Staff Development Handbook

**DOI:** 10.5195/jmla.2018.416

**Published:** 2018-04-01

**Authors:** Danielle Becker

**Affiliations:** Medical Library Services, Hennepin County Medical Center, Minneapolis, MN

Who can forget the movie *Desk Set?* Besides showcasing the wit and chemistry of Katharine Hepburn and Spencer Tracy, its premise also predicted what has become a reality for many libraries throughout the country. The film takes place in 1957 at a television network’s reference library, which is supervised by Bunny Watson (Katharine Hepburn). The network decides to buy two computers: one for processing payroll and one for answering simple questions in the library. To ease the transition and to observe library functions, the network sends Richard Sumner (Spencer Tracy), the man who invented a computer named EMERAC (for Electromagnetic MEmory and Research Arithmetical Calculator), to the library. EMERAC sends the librarians into a frenzy because they are certain they will be replaced by this “electric brain” that only errs when “the human element” makes mistakes (like spelling words incorrectly or asking it questions that force it to “evaluate” a function it cannot perform). However, the purpose of EMERAC is to liberate the librarians’ time for more important work, like research.

At the end of the film, Bunny proves that “the human element” is just what makes their library successful when EMERAC gets overloaded trying to answer the rapid-fire ready-reference phone calls coming in from patrons and proudly declares to her staff, “Let’s show them what people can do!” Spoiler Alert: the librarians keep their jobs (which they thought were surely lost through a pink slip mix-up caused by the payroll EMERAC), and EMERAC proves helpful in basic tasks after all.

Sound familiar? Regardless if you have seen the film or not, you know the feeling of having to compete against the machines (databases, the Internet, etc.) that help us do our work. But, as Bunny points out to her fellow librarians, “no machine can do our jobs,” especially if we continually work toward improving and developing ourselves and our staff. *The Library Staff Development Handbook* by Mary Grace Flaherty is just the book to guide both the seasoned and newbie library supervisor alike.

The book opens with a preface that asserts, “An exceptional library of any time requires exceptional staff, individuals who are enthusiastic, engaged and willing to go the extra mile” (p. xi). The work gives readers different facets of staff development with practical examples, samples, tips, and real-life examples from various libraries. This book includes a table of contents, a list of figures and tables, an index, and a bibliography at the closing of each chapter.

The first chapter introduces the reader to the idea that staff development is a continuous and ongoing process that creates a space for personalities to become interwoven into the institution or organization’s culture. To create a culture where fulfilled staff members thrive, the relationships that individuals have with their direct supervisors; the institutional rules, structures, and assets; and individual employees have to be considered. A first step is to look at how effective and inspirational the leaders are, and this chapter does an excellent job breaking down traits and giving useful anecdotes. This chapter contends that “the most important way a supervisor can empower staff is to cultivate an organizational atmosphere that values open communication, support and mutual respect. In practice, envision the ideal workplace and strive to be the supervisor whom you would like to have” (p. 4). Readers know that with technologies (like EMERAC) come the challenges of library staff being tasked to evolve and adapt, but this has been the case since the printing press. Flaherty claims, “When staff are content and fulfilled, it follows that work processes flow more smoothly and the workplace is more pleasant and productive” (p. 7).

In *Desk Set*, the staff was left out of the transition process when the “electronic brain” was introduced to and integrated into their workflow. In the second chapter, Flaherty lays out how to draft strategic, tactical, operational, and staffing plans and effectively communicate them to not just the organization, but also, as is the case with public libraries, to the community as well. The chapter provides resources to identify specific keys for strategic planning success, sample job descriptions, and tenure information with reading lists for additional information. The next chapter moves into the hiring and recruitment process, with clear-cut steps to recruit, interview, evaluate, and hire the best and the brightest. There is a helpful section on how to identify and target the different advertising outlets that are appropriate for the organization and an easy-to-use job advertisement format. There are also interview tips, rubrics for evaluating interviewees’ responses, and problem-solving skills.

Once soon-to-be exceptional staff members have been hired, how can they be guaranteed that the mechanical brain doesn’t replace them? Chapter four coaches leaders on the different types of motivation (extrinsic and intrinsic) and less positive interactions, including progressive discipline, appeals processes, and exit interviews. Flaherty explains that “staff who are positively engaged with their work and coworkers will likely be more positive, happy, and motivated and demonstrate a commitment to the workplace” (p. 46). She includes ideas about how to motivate staff, tools and methods for measuring staff engagement (and the key drivers fueling staff motivation), and how to keep staff happy. Examples she includes for keeping staff happy are focusing on individual staff members’ strengths and identifying their preference for work style, schedule, and so on. An easily forgotten tool to achieve workplace happiness is to remember to have fun while at work. Flaherty cites that “Individuals who report greater levels of workplace fun have significantly higher levels of job satisfaction, lower emotional exhaustion, and lower emotional dissonance” (p. 54).

The next chapter interprets the term “staff development” to be a continuous, ongoing process that integrates into the overall functions and activities of the institution. Some examples are: education reimbursement, conference participation, allowance for self-directed activities, and ad-hoc workshops. She outlines steps for identifying and determining staff needs for development, such as administering surveys (an example template is provided). She follows with writing a continuing education policy (again, a template is provided) and looks at self-directing learning through online resources and mentoring opportunities. Chapter six explains in detail how to evaluate staff. She provides numerous and varied approaches to this normally stress-evoking process. This chapter focuses on the objectives-based performance review process, where there is staff interaction and engagement. I like this approach because the expectations are clear, performance is measurable, and staff goals can be linked to institutional goals. There are several helpful tools such as sample performance review forms, sample self-evaluations, sample performance, and a review appeals form.

The book wraps up with a chapter on additional funding resources for staff activities and a chapter on how to go forward in a world where “rapidly changing technologies, combined with unprecedented access to all types of information, are causing myriad societal changes” (p. 125). Unlike the characters in *Desk Set* who were ill-informed about the changes happening in their library, to be successful we need ongoing communication. To do our jobs successfully, we need to be partial owners of our institutions’ successes. As Flaherty put it, “If staff are aware of problems and any challenges the organization might be facing, they can be involved in identifying and enacting solutions” (p. 126). Communicating our inside perspective with our supervisors and institutions can help inform and influence change, while progressing our profession. As Bunny exclaimed in *Desk Set*, “Peg, calm down! No machine can do our jobs!” This book inspires me to take “the human element” to the next level and continually develop myself and my staff because Bunny was absolutely right, no machine can do what we do, and I for one, aim to keep it that way.

